# Outdoor particulate matter (PM_10_) exposure and lung cancer risk in the EAGLE study

**DOI:** 10.1371/journal.pone.0203539

**Published:** 2018-09-14

**Authors:** Dario Consonni, Michele Carugno, Sara De Matteis, Francesco Nordio, Giorgia Randi, Martina Bazzano, Neil E. Caporaso, Margaret A. Tucker, Pier Alberto Bertazzi, Angela C. Pesatori, Jay H. Lubin, Maria Teresa Landi

**Affiliations:** 1 Epidemiology Unit, Fondazione IRCCS Ca’ Granda—Ospedale Maggiore Policlinico, Milan, Italy; 2 Department of Clinical Sciences and Community Health, Università degli Studi di Milano, Milan, Italy; 3 National Heart & Lung Institute, Imperial College London, London, United Kingdom; 4 TIMI Study Group, Division of Cardiovascular Medicine, Brigham and Women's Hospital, Harvard Medical School, Boston, MA, United States of America; 5 European Commission, DG Joint Research Centre, Ispra, Varese, Italy; 6 Master Program in Cognitive Sciences and Decision Making, Università degli Studi di Milano, Milan, Italy; 7 Division of Cancer Epidemiology and Genetics, National Cancer Institute, NIH, Bethesda, MD, United States of America; University of Cincinnati College of Medicine, UNITED STATES

## Abstract

**Objective:**

Cohort studies in Europe, but not in North-America, showed an association between exposure to outdoor particulate matter with aerodynamic diameter ≤10 μm (PM_10_) and lung cancer risk. Only a case-control study on lung cancer and PM_10_ in South Korea has so far been performed. For the first time in Europe we analyzed quantitatively this association using a case-control study design in highly polluted areas in Italy.

**Methods:**

The Environment And Genetics in Lung cancer Etiology (EAGLE) study, a population-based case-control study performed in the period 2002–2005 in the Lombardy Region, north-west Italy, enrolled 2099 cases and 2120 controls frequency-matched for area of residence, gender, and age. For this study we selected subjects with complete active and passive smoking history living in the same municipality since 1980 until study enrollment. Fine resolution annual PM_10_ estimates obtained by applying land use regression modeling to satellite data calibrated with fixed site monitor measurements were used. We assigned each subject the PM_10_ average estimates for year 2000 based on enrollment address. We used logistic regression models to calculate odds ratios (OR) and 95% confidence intervals (CI) adjusted for matching variables, education, smoking, and dietary and occupational variables.

**Results:**

We included 3473 subjects, 1665 cases (1318 men, 347 women) and 1808 controls (1368 men, 440 women), with PM_10_ individual levels ranging from 2.3 to 53.8 μg/m^3^ (mean: 46.3). We found increasing lung cancer risk with increasing PM_10_ category (P-value for trend: 0.04). The OR per 10 μg/m^3^ was 1.28 (95% CI: 0.95–1.72). The association appeared stronger for squamous cell carcinoma (OR 1.44, 95% CI: 0.90–2.29).

**Conclusion:**

In a population living in highly polluted areas in Italy, our study added suggestive evidence of a positive association between PM_10_ exposure and lung cancer risk. This study emphasizes the need to strengthen policies to reduce airborne pollution.

## Introduction

In 2016, lung cancer was the leading cause of cancer deaths worldwide (1.7 million deaths) [1]. Outdoor air pollution and particulate matter (PM) in outdoor air pollution in particular, have been recently classified as carcinogenic to humans by an international panel of experts convened by the International Agency for Research on Cancer (IARC), based on sufficient evidence in epidemiological and animal studies and mechanistic data (genetic and related effects) [2, 3]. It has been estimated that 280,000 deaths and 6.2 million DALYs (disability-adjusted life-years) were attributable to ambient particulate matter pollution in 2016 [4].

The IARC evaluation was largely based on studies of long-term residential exposure to outdoor air pollution in terms of PM. Some of those studies (cohort and case-control, ecological studies were not included), were later included in a meta-analysis to calculate the quantitative relationships between the risk of lung cancer and exposure to PM with aerodynamic diameter ≤2.5 μm (PM_2.5_) and ≤10 μm (PM_10_). The meta-analysis included 18 studies (17 cohorts and a case-control) that provided information necessary to estimate the change in lung cancer risk per 10 μg/m^3^ change in PM [5]. The meta-relative risks (RR) were 1.09 (95% confidence interval, CI: 1.04–1.14) and 1.08 (95% CI: 1.00–1.17) per 10 μg/m^3^ for PM_2.5_ and PM_10_, respectively. The meta-analytic estimate for PM_10_ was based on nine cohort studies: five in North America (meta-RR = 1.02, 95% CI: 0.96–1.09) [6–10], three in Europe, (meta-RR = 1.27, 95% CI: 0.96–1.68) [11–13], and one in New Zealand (RR = 1.16, 95% CI: 1.04–1.29) [14]. One of the cohort studies in Europe was a multicenter study (European Study of Cohorts for Air Pollution Effects, ESCAPE) which combined 17 studies (14 with PM_10_ data) from nine countries (eight with PM_10_ data: Austria, Denmark, Greece, Italy, The Netherlands, Norway, Spain, Sweden, and UK; the Spanish cohort had only information on PM_2.5_) and found a hazard ratio (HR) of 1.22 (95% CI: 1.03–1.45) per 10 μg/m^3^ of PM_10_ [13]. Lung cancer subtypes were examined in three studies, two on adenocarcinoma (meta-RR = 1.29. 95% CI: 1.02–1.63) [10, 13] and one on squamous cell carcinoma (RR = 0.84, 95% CI: 0.50–1.40) [13]. Most studies involved the general population and three were performed in occupational categories: trucking industry [8], teachers [9], and nurses [10]. Four studies were limited to women [9, 10, 12, 15] and one to men [8].

Recently, a nationwide cohort study in The Netherlands [16], a study on female lung cancer mortality in Italy [15], and a case-control study from Korea [17] confirmed the positive association between lung cancer risk and PM_10_.

Those studies had one or more limitations, including: lack of incidence data; exposure assessment performed after the follow-up period or a few years before lung cancer occurrence; no or limited information on cigarette smoking; small numbers of lung cancer cases; ecological design. To better characterize the association between lung cancer risk and pollution and to provide more precise risk estimates, more studies are needed.

The aim of our study was to evaluate the association between exposure to outdoor PM_10_ pollution and lung cancer risk within the Environment And Genetics in Lung cancer Etiology (EAGLE) study, one of the largest population-based case-control studies on lung cancer, performed in 2002–2005 in five areas (Milan, Monza, Brescia, Pavia, and Varese) in the Lombardy Region, north-west Italy [18]. EAGLE includes incident lung cancer cases from 13 hospitals covering approximately 80% of lung cancer cases from the catchment area. EAGLE cases and controls have good quality PM_10_ exposure data for the year 2000, which was derived from geocoded annual average PM_10_ estimates for Lombardy based on the application of land use regression techniques to satellite data [19]. Complete and accurate information on potential confounders makes EAGLE very suitable for this analysis.

## Methods

### The EAGLE study

The EAGLE study is a population-based case-control study designed to investigate the genetic and environmental determinants of lung cancer (https://eagle.cancer.gov/). It was conducted in the period from April 2002 to February 2005 in the Lombardy region, north-west Italy, currently 10 million people, which is served by a network of modern hospitals, medical schools, and a regional public health service. The study area included five cities (Milan, Monza, Brescia, Pavia, and Varese) and surrounding towns and villages, for a total of 216 municipalities (over 3 million people). These municipalities had been selected based on preliminary examination of the catchment areas of the 13 participating hospitals (nine located in the Milan area): specifically, we selected only municipalities in which over 80% of lung cancer cases were admitted to one of the 13 hospitals.

The area of Varese is located in a hilly region close to the Pre-Alps. The majority of the municipalities in the other four areas are located in the Po River Valley, one of the most polluted areas in Europe [20]. The city of Milan (currently about 1.3 million residents), is the main in Lombardy. The average of daily PM_10_ levels in Milan over the period 2003–2006 was 52.5 μg/m^3^ (5^th^-95^th^ percentiles 16.2–120.8) [21]. Although PM_10_ concentration has been decreasing in recent years, average annual levels and winter peaks remain considerable [22, 23].

Based on 2001 census data, the population aged 35–79 years in the 216 municipalities included 1,642,074 people (774,764 men, 867,310 women). The number of municipalities (population sizes) in the five areas were as follows: Milan area: 19 (963,341); Monza area: 10 (155,491); Brescia area: 60 (272,786); Pavia: 71 (122,036); Varese: 56 (128,420) [18].

The study was approved by the institutional review boards (IRBs) of the following institutions: USA: National Cancer Institute, Bethesda, MD; Italy: Università degli Studi di Milano, Milan; Istituti Clinici di Perfezionamento, Milan and Fondazione IRCCS Ospedale Maggiore Policlinico, Mangiagalli e Regina Elena, Milan (now Fondazione IRCCS Ca’ Granda Ospedale Maggiore Policlinico, Milan); Ospedale Niguarda, Milan; Istituto Clinico Humanitas, Rozzano; Istituto Scientifico Universitario Ospedale San Raffaele, Milan; Ospedale Luigi Sacco, Milan; AO San Paolo, Milan; AO Ospedale San Carlo Borromeo, Milan; Ospedale Fatebenefratelli e Oftalmico, Milan; Ospedale San Giuseppe, Milan; Ospedale San Gerardo, Monza; Spedali Civili, Brescia; Fondazione IRCCS Policlinico San Matteo, Pavia; Ospedale di Circolo e Fondazione Macchi, Varese. Cases and controls signed an informed consent.

#### Lung cancer cases

Among residents in the selected 216 municipalities we enrolled subjects aged 35–79 years with newly diagnosed primary lung cancer cases of any histological type and stage. The diagnosis of lung cancer was confirmed by pathology reports from surgery, biopsy or cytology samples in approximately 95% of cases, and on clinical history and imaging for the remaining 5%. Pathological (when available) or clinical stage at diagnosis was coded according to the TNM Classification of Malignant Tumors, 7th edition [24]

The response rate (participant/eligible) was 86.6%. The total number of confirmed lung cancer cases was 2101. In the subsequent years we revised the diagnosis for two subjects and excluded them, leaving 2099 cases.

#### Population controls

The population with official residence in the selected 216 municipalities area represented the study pool from which controls were sampled. Information on subjects’ demographics and on their family physicians was contained in the Regional Health Service (RHS) database. We sampled potential controls from updated RHS databases obtained periodically (twice a year) from the Lombardy Region. Subjects’ age was calculated as of pre-specified dates, i.e., July 22^nd^ and January 22^nd^ of each year. Controls were frequency matched to cases for residence (five areas), gender, and five-year age classes in the range 35–79 years. More specifically, they were selected randomly within 90 cells (5×2×9 combinations of area-gender-age) to yield a set of controls with a distribution that initially approximated the case distribution based on lung cancer admissions in year 2000, and subsequently was based on enrolled EAGLE lung cancer cases.

The family physicians of potential controls were contacted (first by letter and then by phone) and asked to provide information about eligibility of the potential study subjects and, if eligible, to contact them to inform them about the study. Eligible controls were then contacted by us, again by letter and phone. When the phone number for the selected individuals could not be found, we searched for the phone numbers of other members of the family identified through contact with the corresponding municipalities, or sent pre-stamped return-cards requesting contact information. Overall, we enrolled 2120 controls, with a participation rate of 72.4%.

#### Data collection

Participants answered a computer-assisted personal interview (CAPI), completed an optical readable self-administered questionnaire (both available at https://eagle.cancer.gov), and donated a blood sample. For lung cancers cases, various types of lung biospecimens (tumoral and normal tissue) were also collected. Patients were usually interviewed during hospital admission. Controls were usually interviewed at home by a dedicated team of nurses who also performed blood drawing. The questionnaires focused on lifetime smoking, including environmental tobacco smoking and types of tobacco smoked, complete work history (year of start and stop, industry, occupation) for jobs held for >6 months, residential history, personal and family medical history, nicotine addiction, dietary habits in the year before diagnosis/enrollment, and reproductive history in women.

Industries and job titles were blindly coded following the International Standard Industrial Classification of All Economic Activities (ISIC, 1971) and the International Standard Classification of Occupations (ISCO, 1968) [25]. Exposure to selected lung carcinogens (including asbestos, quartz dusts, polycyclic aromatic hydrocarbons, diesel motor exhaust, and nickel/chromium compounds) was assessed using a job-exposure matrix [26].

### PM_10_ exposure assessment

We estimated daily PM_10_ levels at each residential address using a hybrid spatial-temporal model that uses land-use regression methodologies and satellite measurements of aerosol optical depth (AOD) at a 10 km resolution, as previously described [19]. Briefly, we used ground PM_10_ measurements from 65 monitoring sites in Lombardy from ARPA Lombardia (Regional Environmental Protection Agency of Lombardy) networks and AOD data from the Moderate Resolution Imaging Spectroradiometer (MODIS) satellites. The model was derived in four stages. The base model (stage 1) consisted of a mixed model for observed PM_10_ monitoring data that contained both fixed and day-specific random effects for the intercept, AOD slopes, and temperature slopes. This stage 1 model was fit to data from each year (2000–2009) separately. To accommodate the fact that daily AOD data missingness is not random, the first stage model incorporates inverse probability weighting (IPW) to potentially prevent bias in the regression coefficient estimates and thus in the resulting estimations. In stage 2 of the model, we estimated PM_10_ concentrations in 10×10 km grid cells without monitors but with available AOD measurements using the stage 1 fit. In stage 3 of the model, we estimated daily PM_10_ concentration levels for all grid cells in the study domain for days when AOD data were unavailable. Using the PM_10_ predictions obtained from the first stage of the model as the response, we fit a model containing a smooth function of latitude and longitude (of the grid cell centroid) and a random intercept for each cell. This is similar to universal kriging, extended to include the mean of the PM_10_ monitors on that day (the average PM_10_ concentrations measured at all the available PM_10_ monitors in the region on each day) and random cell-specific slope. To allow for temporal variations in the spatial correlation, a separate spatial surface was fit for each two-month period of each year. Using this method provides additional information about the concentration in the missing grid cells that simple kriging would not. To provide better estimates in cases in which health outcomes are resolved to the specific longitude and latitude for a given study subject residence (200 by 200 m resolution), we fit a local PM_10_ stage (stage 4) that took the residuals constructed by taking the difference between a given monitored PM_10_ concentration and the 10 x 10 km grid prediction from stage 3 for the grid in which that monitor was located, and regressed these residuals on location-specific predictors of pollution (elevation, distance to major roads, percent of open space, point emissions and area emissions). All stages of the models were internally validated with a 10-fold cross-validation technique.

For this work, we merged the database of EAGLE subjects with the database containing annual PM_10_ estimates (over 1.7 million addresses) using address at enrollment as the merging key. We resolved non-merging records by visual inspection and standardization of addresses in the subjects’ file. Then we attributed to each subject the average annual PM_10_ estimate for year 2000.

### Statistical analysis

In the present work we selected only subjects with complete histories of tobacco smoking and exposure to environmental tobacco smoking. The annual average PM_10_ estimates at residence address in year 2000 (the most remote available to us) are a surrogate of the etiologically relevant exposure occurring many years before diagnosis. For this reason, similarly to other studies [14, 16], we excluded subjects who reported having changed municipality of residence since 1980 until enrollment (2002–2005).

We calculated odds ratios (OR) and 95% confidences intervals (CI) for PM_10_ exposure at enrollment using multivariable unconditional logistic regression. All models were adjusted for (or stratified by) the matching variables to avoid the resulting selection bias introduced by matching in case-control studies [27–31]. We calculated two sets of OR. OR1 was adjusted for residence (five areas), gender, age (5-year classes), education level (none, elementary, middle, high, university), and smoking variables including ever smoked cigarettes, mean-centered pack-years (linear, quadratic, and cubic components), years since quitting (categorical: 0 for never/current smokers; otherwise, 0.5–0.9, 1–1.9, 2–4.9, 5–9.9, 10–19.9, 20–29.9, or 30+ years), ever smoking of other types of tobacco (cigars, cigarillos, pipe), and ever exposed to environmental tobacco smoking (at home in childhood or in adult life at home or at workplace). OR2 was, additionally, adjusted for factors that have been shown to be associated with lung cancer risk in EAGLE and other studies, including consumption of red and processed meat [32], fruit, and vegetables [33] (categorical variables: low, medium, high), and occupational exposure to asbestos, respirable crystalline silica, polycyclic aromatic hydrocarbons, diesel motor exhausts, and nickel/chromium compounds (categorical variables: none, low, high) [26]. To show the effect of smoking adjustment, we also calculated odds ratios adjusted only for area, gender, age, and education (OR0). In supplementary analyses in women, we further adjusted for reproductive variables, including age at first birth (<22, 22–25, 26–30, 30+ years), age at menopause (<46, 46–50, 50+ years), and use of hormone replacement therapy (no, yes) [34].

Average annual PM_10_ exposure was modeled in two ways: 1) categorical, in which we divided PM_10_ in 5 groups according to quintiles among controls. For calculation of P-values for the test of no log-linear trend we used the within category median as the quantitative metric for PM_10_ exposure; 2) continuous log-linear, in which we calculated the OR per 10 μg/m^3^ increase in PM_10_. using individual PM_10_ exposure data. For the main analyses we fit a single regression model for all the five areas.

We performed various sensitivity analyses. First, we calculated odds ratios using four PM_10_ categories. Second, because there were missing data regarding dietary and occupational variables, we generated 100 samples by imputing the missing values based on the rest of the covariates using a multinomial model and then fitted logistic regression models to calculate fully adjusted OR2 based on complete data. Third, we excluded two subjects with low (<20 μg/m^3^) PM_10_ values. Fourth, we excluded subjects with other cancers.

Additional analyses of lung cancer risk and PM_10_ exposure were performed after stratifying by area, gender, and smoking status. When stratifying by area, we performed two sets of analyses: a) considering the Milan area (19 municipalities, including the city of Milan) and the other four areas combined; b) considering the five areas separately. We also analyzed separately the residents in the city of Milan (the largest and most polluted) and the residents in all the other 215 municipalities. Effect modification across those variables was tested by means of Wald tests for interaction (product) terms. Moreover, although we did not *a priori* expect different slopes in the five areas (air quality in Lombardy can be considered quite similar), we also fit random effects models that included random slopes using the approach outlined in DerSimonian and Kacker (2007) [35].

Sub-analyses for the three main histologic types (adenocarcinoma, squamous cell carcinoma, and small cell carcinoma) were also performed using polytomous logistic regression. Homogeneity across histology was assessed using Wald tests.

We do not have individual information relevant to potential radon exposure (e.g., house characteristics, the floor on which participants lived). However, data from ARPA Lombardia shows that there are substantial differences in radon concentration within Lombardy, with values gradually increasing from the Po River Valley towards the hills and mountains (https://goo.gl/m6NhfA). We used a publicly available database that reports, for each municipality in Lombardy the “probability that a generic house at ground floor has a radon concentration higher than 200 Bq/m^3^" (https://goo.gl/oc2bNs). We assigned these probabilities to study participants based on municipality of residence. Being ecological measures, we did not use them in logistic regression models; rather, we calculated their correlation with PM_10_ levels and calculated their averages by area of residence and PM_10_ category to determine the direction of potential confounding by radon.

Statistical analyses were performed with Stata 15 [36].

## Results

Out of 4219 subjects (2099 cases, 2120 controls), all of European descent, enrolled in the EAGLE study in the period 2002–2005, interview data was available for 4060 (1944 cases, 2116 controls). For the present study we eliminated 7 subjects (3 cases and 4 controls) lacking data on active cigarette smoking (1 control without smoking status, 2 cases and 3 controls without pack-years, and 1 case without time since quitting) and 60 subjects (35 cases, 25 controls) with missing information on passive smoking. From the remaining 3993 subjects (1905 cases, 2088 controls) with complete smoking history we excluded 520 subjects (13.0%, 240 cases, 280 controls) who either had changed municipality of residence between 1980 and enrollment (488 subjects, 218 cases, 270 controls) or had incomplete residence history (32 subjects, 22 cases, 10 controls).

The number of subjects included in analysis was therefore 3473 (1665 cases, 1808 controls) with complete active and passive smoking histories who lived in the same municipality from 1980 to enrollment in 2002–2005 (Table 1). The majority of subjects came from the Milan area. Educational level was higher among controls, while current smokers, as expected, were more frequent among cases. The number of pack-years smoked was particularly high in male cases. Smoking of other types of tobacco was rare among women. Exposure to second hand smoking was similar in men and women. About 10 to 20% of subjects had another (previously or newly diagnosed, i.e., diagnosed during the same hospital admission in which lung cancer was diagnosed) primary cancer. The most frequent lung cancer morphology was adenocarcinoma, especially in women, followed by squamous cell carcinoma. In both genders about two-third of cases were in stages III-IV at diagnosis. There were 208 subjects (6.0%, 166 cases and 42 controls) with missing data on at least one dietary variable that did not contributed to the fully adjusted analyses (OR2); among them, 1 case had also missing information on occupational exposure to carcinogens.

**Table 1:** Selected characteristics of lung cancer cases and controls with complete smoking history and who have always been residing in the same municipality since 1980 to enrollment, the EAGLE study, Lombardy, Italy, 2002–2005.

	Men				Women			
	Cases		Controls		Cases		Controls	
	No.	%	No.	%	No.	%	No.	%
**Total**	1318	100	1368	100	347	100	440	100
**Area of residence**								
Milan	871	66.1	964	70.5	258	74.4	318	72.3
Monza	95	7.2	73	5.3	17	4.9	19	4.3
Brescia	159	12.1	156	11.4	37	10.7	48	10.9
Pavia	91	6.9	68	5.0	13	3.7	28	6.4
Varese	102	7.7	107	7.8	22	6.3	27	6.1
**Age (years)**								
Mean (SD)	67.3	(7.6)	66.4	(7.7)	65.5	(9.8)	65.0	(9.5)
**Education level**								
None	82	6.2	54	4.0	17	4.9	23	5.2
Elementary	548	41.6	378	27.6	117	33.7	134	30.4
Middle	353	26.8	387	28.3	117	33.7	139	31.6
High	263	19.9	365	26.7	82	22.6	119	27.0
University	72	5.5	184	13.4	14	4.0	25	5.7
**Cigarette smoking**								
Never	25	1.9	328	24.0	88	25.4	254	57.7
Former (>6 months)	624	47.3	687	50.2	100	28.8	98	22.3
Current	669	50.8	353	25.8	159	45.8	88	20.0
**Cigarette pack-years**								
Mean (SD)	50.6	(28.5)	21.9	(22.4)	24.3	(22.3)	7.3	(14.0)
**Smoking of other types of tobacco (cigars, pipe)**								
Never	1095	83.1	1118	81.7	342	98.6	438	99.6
Ever	233	16.9	250	18.3	5	1.4	2	0.4
**Second hand smoking**								
Never	75	5.7	126	9.2	18	5.2	29	6.6
Ever	1243	94.1	1242	90.8	329	94.8	411	93.4
**Other cancer(s)**^a^								
No	1102	83.6	1240	90.6	281	81.0	396	90.0
Yes	216	16.4	128	9.4	66	19.0	44	10.0
**Lung cancer morphology**								
Adenocarcinoma	495	37.6			189	54.5		
Squamous cell carcinoma	394	29.9			38	11.0		
Large cell carcinoma	49	3.7			25	7.2		
Non-small cell carcinoma	137	10.4			36	10.4		
Small cell carcinoma	136	10.3			34	9.8		
Other	44	3.3			13	3.8		
Not available	63	4.8			12	3.5		
**Lung cancer stage**^**b**^								
In situ	1	0.1			0	0.0		
IA	113	8.6			38	11.0		
IB	124	9.4			40	11.5		
IIA	101	7.7			21	6.0		
IIB	78	5.9			16	4.6		
IIIA	224	17.0			77	22.2		
IIIB	128	9.7			29	8.4		
IV	509	38.6			122	35.2		
Not available	40	3.0			4	1.2		
**PM**_**10**_ **(μg/m**^**3**^**), year 2000**								
Mean (SD)	46.5	(4.5)	46.5	(4.6)	47.0	(4.0)	46.9	(4.1)
Median	47.6		47.7		48.2		47.7	
Percentiles (25^th^ and 75^th^)	44.2	49.9	44.4	49.8	45.3	49.9	44.9	49.9
Min and max	25.6	53.8	2.3	53.8	31.5	53.8	24.2	53.2

^a^Primary cancer(s) (previously or newly-diagnosed) other than lung cancer

^b^Pathological (when available) or clinical stage at diagnosis according to the TNM Classification of Malignant Tumors, 7th edition

**Fig 1:**
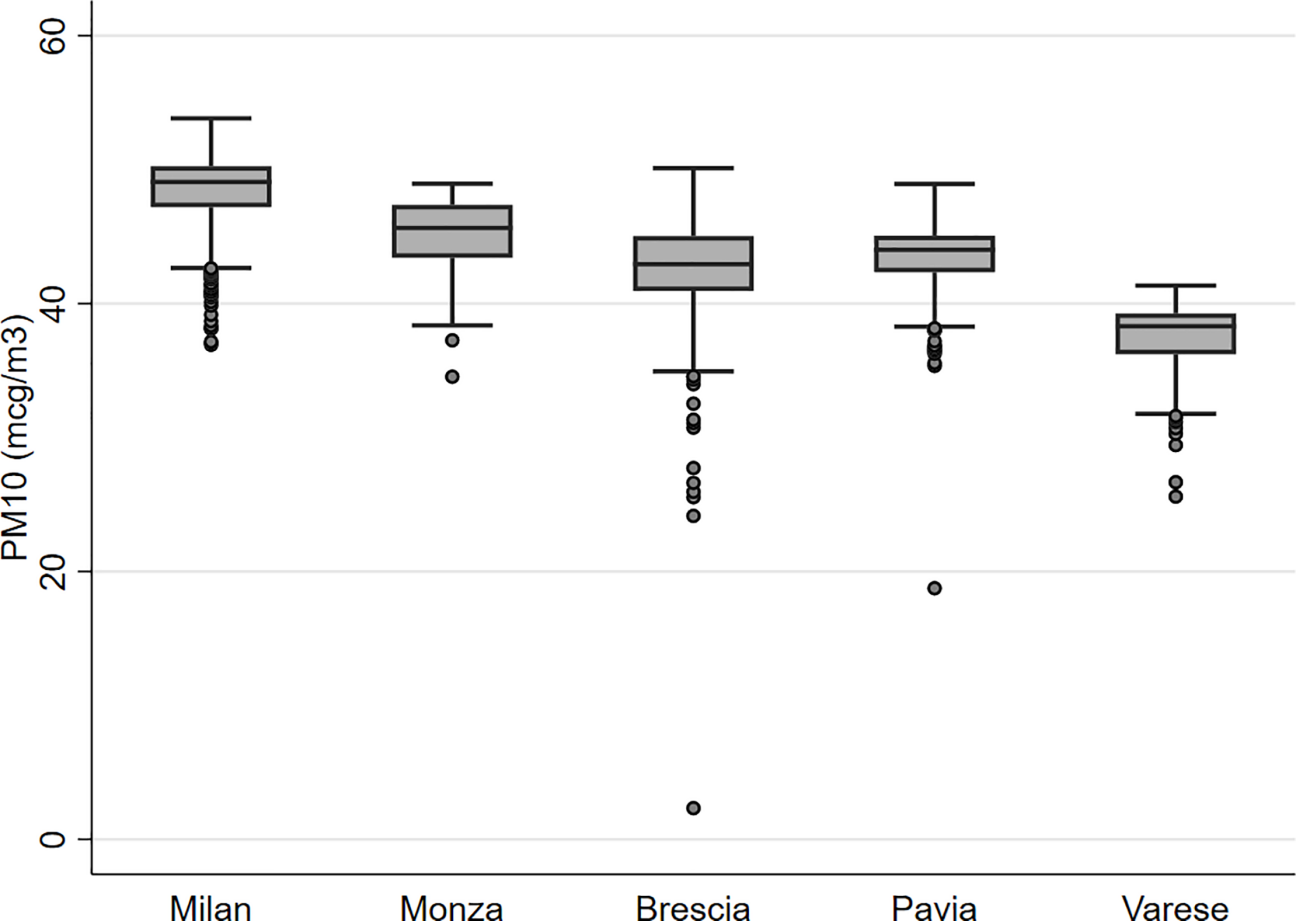
Box plot of PM_10_ concentration levels in year 2000 by area, the EAGLE study, Lombardy, Italy, 2002–2005.

The average PM_10_ concentrations in year 2000 ranged from 37.5 (Varese area) to 48.6 μg/m^3^ (Milan area) (Table 2 and Fig 1). The two lowest individual values in the areas of Brescia (2.3 μg/m^3^) and Pavia (18.7 μg/m^3^) were from two male controls living in two small villages at 1,250 and 729 m of altitude, respectively. The upper PM_10_ category included only subjects living in the Milan area except one, while all subjects from the Varese area fell into the lower category (S1 Table). Overall and after stratification by case status, area, and sex, and among ever smokers, there was no correlation between cigarette pack-years and PM_10_ estimates (Spearman’s rho correlation coefficients ranged between -0.03 and +0.06).

**Table 2:** Average PM_10_ concentrations in year 2000 among subjects in the five areas, the EAGLE study, Lombardy, Italy, 2002–2005.

	Milan	Monza	Brescia	Pavia	Varese	Total
**PM**_**10**_ **(μg/m**^**3**^**)**						
Minimum	36.9	34.5	2.3	18.7	25.6	2.3
25^th^ percentile	47.2	43.4	40.9	42.3	36.2	44.5
Mean	48.6	45.2	42.4	43.2	37.5	46.6
Median	49.1	45.6	42.9	44.0	38.3	47.8
75^th^ percentile	50.2	47.4	45.0	45.1	39.3	49.9
Maximum	53.8	48.9	50.1	48.9	41.3	53.8
SD	2.4	2.7	4.3	3.4	2.8	4.4

The probability estimates of high (>200 Bq/m^3^) exposure to radon were strongly inversely correlated with PM_10_ levels (Spearman’s rho correlation coefficient: -0.63, P<0.0001, either including or excluding the two lowest PM_10_ values). The average probabilities in the 5 areas were 0.004% (Milan), 1.8% (Monza), 8.1% (Brescia), 0.4% (Pavia), and 3.5% (Varese). The average probabilities, respectively for the five PM_10_ categories (from lower to upper), were as follows: 5.3%, 0.9%, 0.3%, 0.04%, and 0.004%.

**Fig 2:**
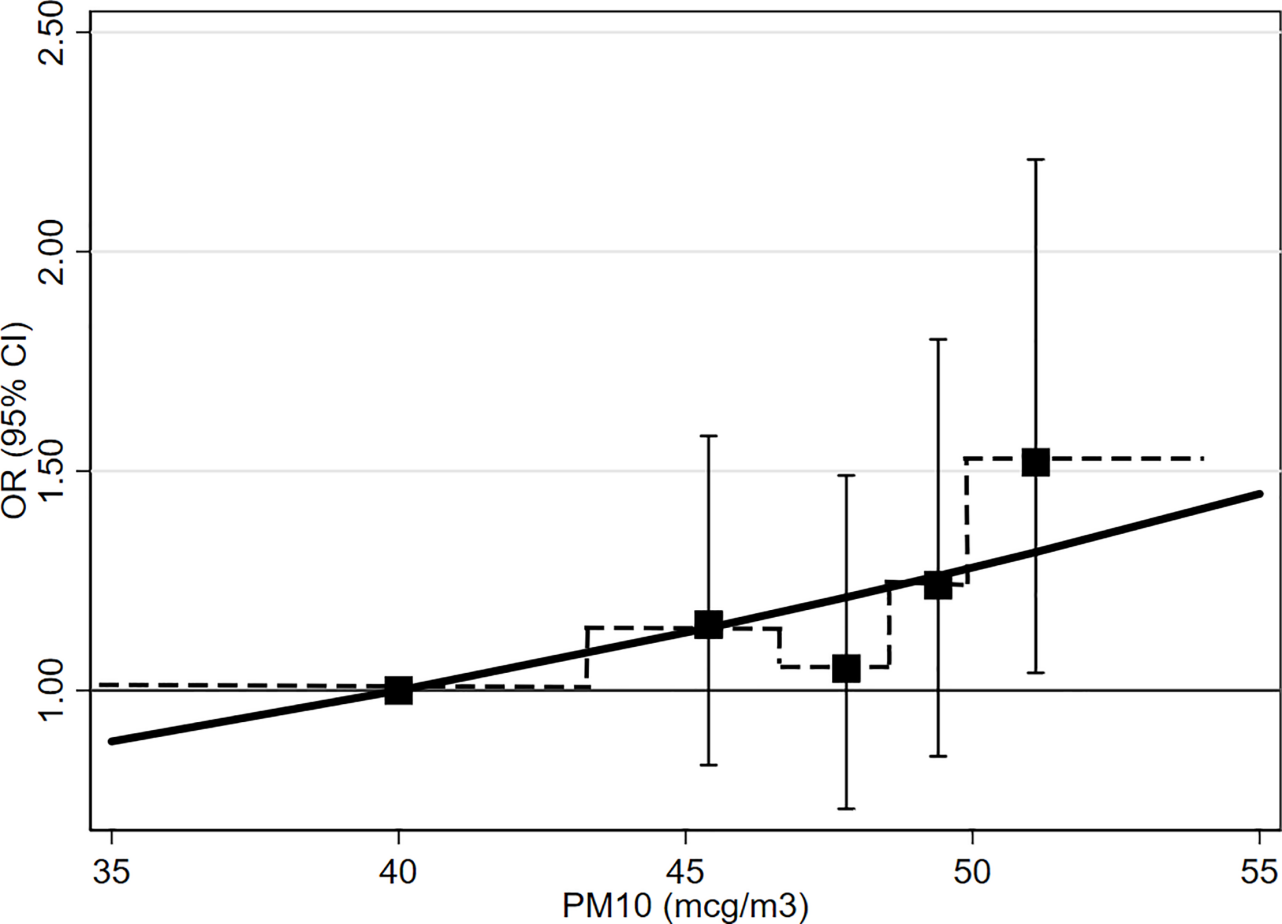
Association between lung cancer risk and average PM_10_ concentration levels in year 2000, the EAGLE study, Lombardy, Italy, 2002–2005. Category-specific odds ratios (OR) and 95% confidence interval (CI) adjusted for area, gender, age, education, smoking (active and passive), diet, and exposure to occupational lung carcinogens, located at the within category medians (solid symbol, dash line) and the fitted log-linear regression with continuous PM_10_, adjusted to pass through the referent category (solid line).

Overall, we found an increasing trend of lung cancer with increasing PM_10_ concentration (P-value for trend for OR2: 0.04), with ORs moderately increased in the 4^th^ (OR2 1.24, 95% CI: 0.85–1.80) and clearly elevated in the 5^th^ PM_10_ category (OR 1.52, 95% CI: 1.04–2.21) (Table 3 and Fig 2). The fully adjusted OR2 per 10 μg/m^3^ was 1.28 (95% CI: 0.95–1.72, P = 0.11). Notwithstanding the absence of (crude) correlation between cigarette pack-years and PM_10_ estimates, active smoking, for which we had previously documented high ORs in both men and women [37, 38], behaved as a positive confounder: smoking-adjusted categorical odds ratios (OR1) were lower than non-adjusted ones (OR0) and the smoking-unadjusted risk excess per 10 μg/m^3^ was 76% (= [0.30–0.17]/0.17×100) upward biased (Table 3). Conversely, dietary and occupational variables acted as negative confounders: fully adjusted OR2 were higher than those adjusted only for area, gender, age, education, and smoking (OR1). Results obtained using four PM_10_ categories showed monotonically increasing ORs and a P-value for trend for OR2 of 0.015 (S2 Table and S1 Fig).

**Table 3:** Lung cancer risk according to average PM_10_ exposure in year 2000, the EAGLE study, Lombardy, Italy, 2002–2005.

PM_10_ category—median (μg/m^3^)	No. cases	No. controls	OR0	95% CI	OR1	95% CI	OR2	95% CI
1–40.0	331	362	1.00	Reference	1.00	Reference	1.00	Reference
2–45.4	361	363	1.28	0.99–1.67	1.12	0.83–1.52	1.15	0.83–1.58
3–47.8	293	359	1.14	0.85–1.53	1.01	0.72–1.42	1.04	0.73–1.49
4–49.4	324	362	1.38	1.02–1.87	1.23	0.86–1.75	1.24	0.85–1.80
5–51.1	356	362	1.50	1.11–2.04	1.39	0.97–1.99	1.52	1.04–2.21
OR per 10 μg/m^3^			1.30	1.02–1.66	1.17	0.88–1.55	1.28	0.95–1.72

OR0, odds ratios adjusted for area, gender, age, and education; OR1, odds ratios additionally adjusted for smoking (active and passive); OR2, odds ratios additionally adjusted for dietary and occupational variables. The ORs per 10 μg/m^3^ were derived from models with continuous PM_10_ concentration levels.

The results (OR2) obtained after multiple imputation of missing values for diet and occupation were as follows: category 2, OR 1.19 (95% CI: 0.87–1.62), category 3, OR 1.05 (95% CI: 0.74–1.48), category 4, OR 1.30 (95% CI: 0.90–1.86), and category 5, OR 1.50 (95% CI: 1.04–2.16); P-value for trend was 0.03; OR2 per 10 μg/m^3^ of PM_10_ was 1.27 (95% CI: 0.95–1.70). When we excluded the two lowest PM_10_ values, the OR2 from categorical analysis were similar (not shown), P-value for trend for OR2 was 0.04, and OR2 per 10 μg/m^3^ of PM_10_ was 1.23 (95% CI: 0.90–1.67). Exclusion of subjects with other cancers yielded the following OR2: category 2, OR 1.12 (95% CI: 0.80–1.58), category 3, OR 1.04 (95% CI: 0.71–1.52), category 4, OR 1.18 (95% CI: 0.79–1.77), and category 5, OR 1.42 (95% CI: 0.95–2.13); P-value for trend was 0.11; OR2 per 10 μg/m^3^ of PM_10_ was 1.33 (95% CI: 0.96–1.85).

When we examined subgroups (S3 Table), in men we found a positive increasing risk (P-value for trend for OR2: 0.03) and a markedly elevated OR2 in the upper category. In women the pattern was less clear, even after further adjustment for reproductive variables (results not shown). The P-values for PM_10_-gender interaction (OR2) were 0.26 (categorical model) and 0.52 (log-linear model).

We found higher ORs for subjects living in the Milan area (P-value for trend for OR2: 0.01) compared with the other four areas combined, but the estimates were imprecise due to the low number of subjects in the lower category of PM_10_ exposure. In fact, the P-values for PM_10_-area interaction (OR2) were high (0.96 from the categorical model and 0.30 from the log-linear model).

Considering the five areas separately, the fully adjusted ORs per 10 μg/m^3^ in the other four areas were as follows: Monza: OR 2.83 (95% CI: 0.54–14.8); Brescia: OR 0.98 (95% CI: 0.52–1.81); Pavia: OR 2.30 (95% CI: 0.61–8.76); Varese: OR 1.05 (95% CI: 0.25–4.45).The fixed-effect P-values for the interaction between PM_10_ and area (OR2) were 0.64 (categorical model) and 0.67 (log-linear model). Moreover, we found no indication of different random slopes in the five areas. In fact, using various alternative formulations the Q-test for homogeneity generally resulted in P-values around 0.20; in one instance we found I^2^ = 0.34 (the proportion of variance due to heterogeneity), while in another τ^2^ was negative and I^2^ could not be determined (i.e., data were not compatible with a random effects modeling).

The ORs were higher (P-value for trend for OR2: 0.001) among residents in the main city of Milan (84.3% of the resident in the Milan area, 937 cases and 1095 controls). The P-values for interaction (OR2) were 0.31 from the categorical model and 0.04 from the log-linear model.

Among former and current smokers there were suggestive positive lung cancer risk trend (P-values for trend for OR2: 0.10 and 0.28, respectively), while in never smokers we found elevated ORs in all categories from 2^nd^ to 5^th^, but with wide confidence intervals. The P-values for PM_10_-smoking interaction (OR2) were 0.86 (categorical model) and 0.97 (log-linear model).

There was a slightly positive trend for adenocarcinoma (P-value for trend for OR2: 0.45) and a monotonic increasing trend for squamous cell carcinoma (P-value for trend for OR2: 0.09), while the OR for small cell carcinoma was elevated only in the 5^th^ category (S3 Table). The P-values for OR2 homogeneity across histology were 0.79 (categorical model) and 0.31 (log-linear model).

## Discussion

This study provides suggestive evidence of a positive association between lung cancer risk and PM_10_ exposure in the population of the EAGLE study, performed in highly polluted areas of the Lombardy Region, north-west Italy, after controlling for other known risk factors for lung cancer. The positive association appeared to be more evident among men (who represented three-fourths of the sample) and for squamous cell carcinoma.

### Strengths and limitations

The strengths of the EAGLE study are the enrollment of incident cases and randomly sampled population controls, the large sample size (particularly for men and for the main lung cancer subtypes), the high quality of clinical documentation [39], and the availability of detailed information on the most important risk factors for lung cancer. Participation rates were good, but slightly lower in controls. Therefore, to exclude possible selection effects from differential participation of cases and controls according to socio-economic status, we adjusted all models for education [40]. The numbers of subjects excluded because of lack of information on smoking data was small (No. 67). Therefore, we can confidently exclude important impacts of these exclusions on relative risk estimates. The fraction of subjects not included in fully adjusted models because of missing data for dietary or occupational variables was small (No. 208). Moreover, additional multiple imputation analyses using all observations found similar results. In summary, we think the study results were not affected by important selection biases.

We were able to adjust for several smoking variables based on complete active and passive smoking history and for several other potential confounding factors, including diet and several occupational carcinogens for which we had previously found clear associations with lung cancer [26, 32, 33, 37, 38, 41, 42]. Moreover, since area of residence was a matching variable, we adjusted for it by introducing fixed effects to ensure complete control [27–29]. When we fit random effects models that included random slopes [35], we found no indication that suggested the need for the random effects approach, which is expected since pollution in the Lombardy region is rather homogeneous. Although we did not have individual data on radon, we could exploit available ecological information on the probability of high (>200 Bq/m^3^) radon exposure to document the marked inverse relationship between PM_10_ and radon estimates and thus conclude that confounding by potential radon exposure is negative (i.e., the expectation is that radon-adjusted relative risks would be higher than non-adjusted ones).

Another strength of the present study is the methodology for creating PM_10_ estimates. The use of satellite data calibrated with monitor measurement and land-use regression models allowed estimation of PM_10_ concentrations at a fine resolution for the whole Lombardy territory.

The major limitation in this study is the lack of data on pollution levels in a period relevant to the pathogenesis of lung cancer (i.e., some 20–30 years before enrollment). The PM_10_ estimates for year 2000 that we used are the oldest data available for the whole Lombardy territory at a fine resolution. Until the late 90’s there were a few monitoring station (mainly located in Milan city) that measured particulate matter as total suspended particles (TSP): the available data indicate that pollution levels in Milan city were about 3 times higher in the 80’s and roughly 1.5–2 times higher in the first half of the 90’s (http://www.arpalombardia.it/qariafiles/varie/MI_PM10.png). Unfortunately, there are no data before 2000 regarding the other areas involved in this study. We previously reported that in the period 2003–2006 the PM_10_ levels in the cities of Milan, Brescia, Pavia, and Varese were 52.5, 49.4, 44.4, and 29.6, respectively; in the years between 2007 and 2014 Milan was always the most polluted city (except in 2014, when Pavia showed the highest level), while Varese was always the least polluted; Brescia had levels similar to those recorded in Pavia (in some years higher in Brescia, in others higher in Pavia) [22]. Finally, the city of Monza, being very close to Milan (<20 km), tends to have pollution levels similar to Milan. In summary, it is plausible that comparable pollution rankings were present before 2000 in the Milan, Monza, and Varese areas, while uncertainties exist about relative levels in Brescia and Pavia.

The second major limitation is the lack of historical information on exact residential address. We tried to partially address this problem by excluding subjects who reported at interview to have resided in different municipalities after 1980, but we do not know how many subjects among those who did not change municipality were in fact resident at the same exact address.

The consequence of these two limitations would be a misclassification of the etiologically relevant exposure. In any case, this limitations would hardly explain the positive association with PM_10_ levels, since we would in principle expect (on average) non-differential misclassification, which most frequently biases results towards the null.

A further limitation of our study is that the only available pollutant was PM_10_. Other pollutants (e.g., NO_x_ and PM_2.5_) started to be routinely measured by monitoring stations of ARPA Lombardia only in more recent years and only at a coarse geographical resolution. However, our findings are indirectly relevant to PM_2.5_: in fact, the average PM_2.5_/PM_10_ ratio in the city of Milan in most recent years was roughly 0.7 [23].

### Comparison with published research

Before our study, a recent large meta-analysis and three additional studies were published on PM_10_ and lung cancer risk. We provide here a synthetic description of each study and highlight their strengths and limitations. The meta-analysis included nine cohort studies (three from Europe, five from the USA, and one from New Zealand) which provided quantitative relative risk estimates of lung cancer (per 10 μg/m^3^ of PM_10_) [5]. The additional studies were conducted in The Netherlands (cohort study) [16], Italy (ecologic study) [15], and South Korea (the only case-control study published so far) [17].

#### European studies

The OR per 10 μg/m^3^ of PM_10_ of 1.28 (95% CI: 0.95–1.72) in our study is in agreement with those of three other European cohort studies [11–13] included in the quoted meta-analysis (meta-RR 1.27, 95% CI: 0.96–1.68) [5]. Among these studies, one was performed in England, one in Germany, while the ESCAPE study was a multicenter cohort study.

The English study consisted of a large cohort of more than 830,000 general practice patients followed-up for mortality in the period 2003–2007 [11]. PM_10_ in the year 2002, estimated from emission-based models, ranged from 12.6 to 29.8 (mean: 19.7). The HR per 10 μg/m^3^, adjusted for smoking (smoking status and cigarettes per day), and income (census deprivation score) was 1.03 (95% CI: 0.88–1.21) based on 5244 lung cancer deaths.

The German study was a small cohort study of 4800 women recruited from 1985 to 1994 followed-up for mortality through 2008 [12]. PM_10_ in the period 1985–1994 was derived from TSP measured by fixed site monitors using the formula PM_10_ = TSP×0.71. Minimum-maximum PM_10_ values were 34.8–52.5 μg/m^3^ (mean: 43.7). Based on 41 lung cancer deaths, the HR adjusted for education and smoking status per 10 μg/m^3^ was 2.39 (95% CI: 1.35–4.22). Smoking status included information on pack-years and passive smoking.

The large (about 313,000 subjects) multicenter ESCAPE study pooled 14 cohorts with PM_10_ data from eight countries (one cohort in Norway, Denmark, UK, Austria, and Greece; four cohorts in Sweden; two cohorts in The Netherlands; three cohorts in Italy). The enrollment years ranged from 1985 (minimum) to 2005 (maximum) [13]. Air pollution concentrations at the baseline residential addresses of study participants were estimated (back-extrapolated) by land-use regression models applied to pollution data collected in 2008–2011 (i.e., after the study period). Another limitation of the ESCAPE study (shared by our study) was that information on residential address was available only in some cohorts. Back-extrapolated PM_10_ levels in the ESCAPE study ranged from about 5 to about 90 μg/m^3^ (mean: 21.3), with the highest levels in South Europe (Italy and Greece, medians above about 35 μg/m^3^). Based on 1931 lung cancer cases, the HR adjusted for a number of variables measured at enrollment (no update was performed), including active and passive smoking, socio-economic status, fruit intake, and occupation was 1.22 (95% CI: 1.03–1.45) per 10 μg/m^3^. The analysis restricted to participants who did not changed residence during follow-up yielded a HR of 1.48 (95% CI: 1.16–1.88, based on 893 cases from 10 cohorts).

Pollution levels in the EAGLE study are similar to those in Athens, Greece, and Turin, Italy, the latter city also located in the highly polluted Po River Valley [20]. The lung cancer estimates for these two cities were higher than the overall HR in the ESCAPE study (EPIC-Athens, RR 1.55 (95% CI: 1.00–2.40, 18 cases); EPIC-Turin, RR 1.45 (95% CI: 0.69–3.04, 48 cases), SIDRIA-Turin, RR = 1.41, 95% CI: 0.46–4.31, 19 cases).The ESCAPE study found ORs for adenocarcinoma (based on 663 cases) and squamous cell carcinoma (322 cases) respectively of 1.51 (95% CI: 1.10–2.08) and 0.84 (95% CI: 0.50–1.40) per 10 μg/m^3^ of PM_10_. Among subjects who did not move the HRs were 2.27 (95% CI: 1.32–3.91, 329 cases from eight cohorts) for adenocarcinoma and 0.64 (95% CI: 0.28–1.48, 132 cases from three cohorts) for squamous cell carcinoma. In our EAGLE study, based on larger numbers, there was a steeper relationship for squamous cell carcinoma (fully adjusted OR2 1.44, 95% CI: 0.90–2.29) than for adenocarcinoma: (OR2 1.13, 95% CI: 0.79–1.72) (S3 Table). It should be noted that the histology-specific ESCAPE estimates were largely based on Scandinavian countries and Austria; only one Italian cohort (EPIC-Turin: 28 cases with adenocarcinoma) could be included in analyses, while none of the Italian cohorts contributed squamous cell carcinoma cases.

After publication of the meta-analysis, the nation-wide Dutch Environmental Longitudinal Study (DUELS) was completed. A cohort of more than 7 million people selected in 2004 living at the same residential address since 1999 was followed-up for mortality through 2011. Median PM_10_ concentration in year 2001, calculated using land use regression methods, was 29 μg/m^3^ (5^th^-95^th^ percentile: 24–32). Based on 53,735 lung cancer deaths, a HR of 1.26 (95% CI: 1.21–1.30) per 10 μg/m^3^ was calculated, almost identical to the OR calculated in our study [16]. The HR was adjusted for marital status, region of origin, standardized household income, and neighborhood (postal digit) social status. No information on smoking and diet was available.

An ecological mortality study (2000–2011, 24,149 lung cancer deaths) was recently performed in Italy among women living in 64 province capital city municipalities [15]. Annual PM_10_ average estimates (ranging from 15 to 44 μg/m^3^) were obtained from fixed monitoring sites. A standardized mortality rate increase (standard population: 2001 Italian census) per 10 μg/m^3^ increment of PM_10_, adjusted for percentage of smokers and deprivation index, of 3.25 per 100,000 person-years was found. Smoking data were extrapolated from a survey of a random sample of Italian families in 2015.

#### USA studies

Five US studies contributed to a meta-analysis on lung cancer and PM10, with an overall meta-RR of 1.02 (95% CI: 0.96–1.09) [5].

The first study on PM_10_ and lung cancer in the meta-analysis was the Adventist Health Study on Smog (ASHMOG), in which a cohort of 6338 nonsmoking Californian adults was followed-up for cancer incidence from 1977 to 1992 [6]. PM_10_ levels (range: 0–85, mean: 51.0 μg/m^3^) was measured through fixed-site monitoring stations from 1973 to 1992. Until 1987, PM_10_ was derived from TSP. Information on education, fruit consumption, smoking (active and passive), residential, medical, and occupational history was collected at baseline (1977) and updated in 1987 and 1992. In men only (2278 subjects, 16 lung cancer cases) the HR was 5.21 (95% CI: 1.94–13.99) for an interquartile range of 24 μg/m^3^ of PM_10_. From these estimates of HR and confidence limits we calculated, using the formula exp[ln(estimate)/2.4] a HR per 10 μg/m^3^ of 1.99 (95% CI: 1.32–3.00). We obtained the same results using the Cox regression coefficient (ln(HR) = 0.068759) and standard error (0.02101) reported in Table 4 of the paper (which are per 1 μg/m^3^) [6]. Hence, we think the RR of 1.16 (95% CI: 1.02–1.32) for this study reported in Fig 1B of the meta-analysis paper is incorrect [5].

A cohort study performed by the American Cancer Society as part of the Cancer Prevention Study (ACS-CPS-II) examined the relationship between air pollution and mortality among 415,000 people enrolled in 1982–1998. Fixed site monitors were used to estimate exposure to TSP (until the late 1980s) and PM_10_ (available in the early to mid-1990s). Mean PM_10_ concentration in 1982–1998 was 28.8 μg/m^3^ [7]. The meta-analysis reported an RR of 0.98 (95% CI: 0.95–1.01) per 10 μg/m^3^ of PM_10_, adjusted for education, smoking, and dietary and occupational variables [5]. We note that the most results in the original paper regarded PM_2.5_, however it was not included in the calculation of meta-RR for PM_2.5_ in the meta-analysis paper [5]. We were able to find only one RR (slightly greater than 1.00) for PM_10_ and lung cancer in the period 1987–1996 in Fig 5C of the original paper [7].

A cohort study in the USA examined the mortality (1985–2000) of employees with occupational exposure to vehicle exhaust, the Trucking Industry Particle Study (TrIPS) [8]. Annual average exposures to PM_10_ were determined for 1985 through 2000 from a model using spatial smoothing and geographic information system-based covariates. Average PM_10_ exposure during the study period was 26.8 μg/m^3^. The analyses were performed among about 40,000 drivers who returned daily to their homes. Based on 475 lung cancer deaths, a HR of 1.08 (95% CI: 0.91–1.30) per 10 μg/m^3^ was calculated, based on last known address and adjusted for occupational exposures. No information on smoking was available in this study.

The California Teachers Study (TCS) was a cohort of female public-school professionals investigated for the association between PM_10_ and lung cancer mortality in the period 1997–2005 [9]. The analysis was restricted to women living in California when they joined the study. PM_10_ estimates 1996–2005 (range: 9.19 to 82.64 μg/m^3^; mean: 29.21) were obtained by combining fixed-site monitors measurements and inverse-distance weighting interpolations. The number of lung cancer deaths observed among more than 61,000 women was 275, with a HR, adjusted for education, smoking (active and passive), and for dietary fat, fiber, and calories of 0.93 (95% CI: 0.81–1.07) per 10 μg/m^3^.

Finally, the Nurses’ Health Study (NHS) cohort study examined the association between PM_10_ estimated with spatio-temporal models and lung cancer incidence among about 103,000 female nurses in the period 1994–2010 [10]. PM_10_ ranged from 3.17 to 74.79 μg/m^3^. (mean: 21.6). The analyses, adjusted for income, smoking (active and passive), and dietary variables, yielded a HR of 1.04 (95% CI: 0.95–1.14) per 10 μg/m^3^ of PM_10_, based on 2155 lung cancer cases. The HR for adenocarcinoma was 1.18 (95% CI: 0.97–1.45, 847 cases), which increased to 1.41 (95% CI: 0.95–2.09, 425 cases)) when the analysis was restricted to never or former smokers who had quit at least 10 years previously; due to small numbers, no analysis on squamous cell carcinoma was performed [10]. In this study the exposure metrics was the time-varying cumulative PM_10_ average experienced in the previous 72 months.

#### New Zealand study

In the New Zealand Census-Mortality Study the records of the entire 1996 resident population (3.7 million people) were probabilistically linked to mortality data 1996–1999 [14]. Average PM_10_ exposure at 1996 census, estimated using land use regression models, was 8.3 μg/m^3^ (range: 0 to 30, approximately). Exposure was divided based on quintiles, then the first and second category were combined. The analyses were restricted to about 1 million people living in urban areas. The number of lung cancer deaths in the study period was 1683. Using the averages of the four PM_10_ categories (0.1, 7, 14, and 19 μg/m^3^, respectively), the OR per 10 μg/m^3^ of PM_10_, adjusted for ethnicity, social deprivation, income, education, and smoking status among subjects who had not changed residence since the 1991 census was 1.16 (95% CI: 1.04–1.29) per 10 μg/m^3^.

#### South Korea study

The only case-control study on lung cancer which calculated quantitative relative risk estimates (per 10 μg/m^3^ of PM_10_) we are aware of was recently performed in South Korea [17]. The study population consisted of 908 histologically confirmed incident lung cancer cases admitted to three university hospitals in Seoul and Incheon and 908 individually matched population-based controls (years 2014–2105). Compared to our study, the strengths of this study were the PM_10_ exposure assessment performed using land use regression models in a 20-year period before subjects’ enrollment and the complete reconstruction of residential histories from 1995 onward. PM_10_ ranged from 18.25 to 95.98 μg/m^3^ (mean: 55.27) and the OR adjusted for education, smoking, fruit consumption, and occupational exposure to carcinogens was 1.09 (95% CI: 0.96–1.23) per 10 μg/m^3^ of PM_10_. The association was slightly stronger for squamous cell carcinoma (OR = 1.15, 95% CI: 0.98–1.35, 188 cases) than for adenocarcinoma (OR = 1.09, 95% CI: 0.97–1.22, 559 cases). The highest OR for small cell carcinoma was 1.36 (95% CI: 1.05–1.73, 55 cases) which well compares with our fully-adjusted estimate of 1.35, 95% CI: 0.70–2.63). No increase was found for large-cell carcinoma (OR 0.87, 95% CI: 0.60–1.25, 35 cases). Compared to the EAGLE study, a limitation of this study was the crude information on smoking. In fact, only smoking status (never, former, current) was available, and information on passive smoking concerned only the week before enrollment.

In summary, the studies on the association between PM_10_ and lung cancer risk discussed above had several limitations. Most studies were based on mortality data [7–9, 11, 12, 14, 16].This implies that the period after cancer occurrence, although etiologically irrelevant, is included in the analysis. Moreover, no analysis by histologic type was reported. In one study [13] the exposure assessment was performed after the follow-up period. In other studies the exposure assessment was too close to the outcome occurrence [9, 10, 14, 16], similar to our study. In other studies limited or no information on smoking was available [8, 17]. Two studies were based on a small number of lung cancer cases [6, 12], as were the three Italian cohorts included in the large multicenter ESCAPE study [13]. Finally, one was an ecological study, included here just for completeness [15].

## Conclusions

In a population living in highly polluted areas in north-west Italy we found suggestive evidence of a positive association between outdoor particulate matter and risk of lung cancer, after controlling for most potential confounders. Our study support previous findings even in a different context as the Lombardy region of Italy, where airborne particles have been associated to a significant impact on various health outcomes [21–23, 43]. Future studies including data on long-term pollutant levels are necessary to precisely estimate the strength of this association. Our study underlines the need to strengthen policies to reduce airborne pollution in the Po River Valley, one of the most polluted areas in Europe.

### Ethics approval and consent to participate

The study was approved by the institutional review boards (IRBs) of the following institutions: USA: National Cancer Institute, Bethesda, MD; Italy: Università degli Studi di Milano, Milan; Istituti Clinici di Perfezionamento, Milan and Fondazione IRCCS Ospedale Maggiore Policlinico, Mangiagalli e Regina Elena, Milan (now Fondazione IRCCS Ca’ Granda Ospedale Maggiore Policlinico, Milan); Ospedale Niguarda, Milan; Istituto Clinico Humanitas, Rozzano; Istituto Scientifico Universitario Ospedale San Raffaele, Milan; Ospedale Luigi Sacco, Milan; AO San Paolo, Milan; AO Ospedale San Carlo Borromeo, Milan; Ospedale Fatebenefratelli e Oftalmico, Milan; Ospedale San Giuseppe, Milan; Ospedale San Gerardo, Monza; Spedali Civili, Brescia; Fondazione IRCCS Policlinico San Matteo, Pavia; Ospedale di Circolo e Fondazione Macchi, Varese.

## Supporting information

S1 Table Distribution of subjects across area and categories of average PM_10_ in year 2000, the EAGLE study, Lombardy, Italy, 2002–2005. (DOCX)

S2 Table Lung cancer risk according to average PM_10_ exposure (four categories) in year 2000, the EAGLE study, Lombardy, Italy, 2002–2005. (DOCX)

S3 Table Lung cancer risk according to average PM_10_ exposure in year 2000 by selected variables, the EAGLE study, Lombardy, Italy, 2002–2005. (DOCX)

S4 Table Lung cancer risk according to average PM_10_ exposure in year 2000 by histological type, the EAGLE study, Lombardy, Italy, 2002–2005. (DOCX)

S1 Fig Association between lung cancer risk and average PM_10_ concentration levels (four categories) in year 2000, the EAGLE Study, Lombardy, Italy, 2002–2005. (TIF)

S1 File Minimal anonymized dataset. (XLSX)

S2 File Legend for the minimal anonymized dataset (S1 File). (DOCX)

## Abbreviations

AOD: aerosol optical depth (AOD)
ARPA: Regional Environmental Protection Agency
CI: confidence interval; EAGLE: Environment And Genetics in Lung cancer Etiology
OR: odds ratio
PM_10_: particulate matter with aerodynamic diameter ≤10 μm
