# Supplementary material for: Outdoor particulate matter (PM10) exposure and lung cancer risk in the EAGLE study
Source: PLoS One. 2018 Sep 14;13(9):e0203539. doi: 10.1371/journal.pone.0203539 (PMC6157824; doi:10.1371/journal.pone.0203539)
Supplement: S1 Table — (DOCX) [file pone.0203539.s001.docx]

**S1 Table. Distribution of subjects across area and categories of average PM_10_ in year 2000, the EAGLE study, Lombardy, Italy, 2002-2005.**

|  |  |  | **Milan** | **Monza** | **Brescia** | **Pavia** | **Varese** | **Total** |
| --- | --- | --- | --- | --- | --- | --- | --- | --- |
| **PM_10_ category** | **Mean** | **Median** | **No.** | **No.** | **No.** | **No.** | **No.** | **No.** |
| **(min-max µg/m^3^)** | **(µg/m^3^)** | **(µg/m^3^)** | **(%)** | **(%)** | **(%)** | **(%)** | **(%)** | **(%)** |
| 1 (2.3-43.5) | 39.4 | 40.0 | 75 | 54 | 224 | 82 | 258 | 693 |
|  |  |  | (3.1) | (26.5) | (56.0) | (41.0) | (100) | (20.0) |
| 2 (43.5-46.8) | 45.3 | 45.4 | 410 | 85 | 128 | 101 | 0 | 724 |
|  |  |  | (17.0) | (41.7) | (32.0) | (50.5) |  | (20.8) |
| 3 (46.8-48.6) | 47.7 | 47.8 | 544 | 52 | 44 | 12 | 0 | 652 |
|  |  |  | (22.6) | (25.5) | (11.0) | (6.0) |  | (18.8) |
| 4 (48.6-50.0) | 49.4 | 49.4 | 665 | 13 | 3 | 5 | 0 | 686 |
|  |  |  | (27.6) | (6.4) | (0.8) | (2.5) |  | (19.8) |
| 5 (50.0-53.8) | 51.1 | 51.1 | 717 | 0 | 1 | 0 | 0 | 718 |
|  |  |  | (29.7) |  | (0.2) |  |  | (20.7) |
| Total (2.3-53.8) | 46.6 | 47.8 | 2411 | 204 | 400 | 200 | 258 | 3473 |
|  |  |  | (100) | (100) | (100) | (100) | (100) | (100) |
