# Supplementary material for: Outdoor particulate matter (PM10) exposure and lung cancer risk in the EAGLE study
Source: PLoS One. 2018 Sep 14;13(9):e0203539. doi: 10.1371/journal.pone.0203539 (PMC6157824; doi:10.1371/journal.pone.0203539)
Supplement: S2 Table — (DOCX) [file pone.0203539.s002.docx]

**S2 Table. Lung cancer risk according to average PM_10_ exposure (four categories) in year 2000, the EAGLE study, Lombardy, Italy, 2002-2005**

| **PM_10_ category - median, min-max (µg/m^3^)** | **No. cases** | **No. controls** | **OR0** | **95% CI** | **OR1** | **95% CI** | **OR2** | **95% CI** |
| --- | --- | --- | --- | --- | --- | --- | --- | --- |
|  |  |  |  |  |  |  |  |  |
| 1 - 41.1; 2.3-44.5 | 426 | 450 | 1.00 | Reference | 1.00 | Reference | 1.00 | Reference |
| 2 - 46.3; 44.5-47.7 | 393 | 454 | 1.09 | 0.86-1.38 | 1.03 | 0.78-1.36 | 1.11 | 0.83-1.48 |
| 3 - 48.8; 47.7-49.8 | 405 | 452 | 1.22 | 0.93-1.59 | 1.15 | 0.84-1.56 | 1.20 | 0.87-1.66 |
| 4 - 50.8; 49.8-53.8 | 441 | 452 | 1.41 | 1.08-1.86 | 1.35 | 0.99-1.86 | 1.52 | 1.09-2.12 |

OR0, odds ratios adjusted for area, gender, age, and education; OR1, odds ratios additionally adjusted for smoking (active and passive); OR2, odds ratios additionally adjusted for dietary and occupational variables.
