# Supplementary material for: Outdoor particulate matter (PM10) exposure and lung cancer risk in the EAGLE study
Source: PLoS One. 2018 Sep 14;13(9):e0203539. doi: 10.1371/journal.pone.0203539 (PMC6157824; doi:10.1371/journal.pone.0203539)
Supplement: S3 Table — (DOCX) [file pone.0203539.s003.docx]

**S3 Table. Lung cancer risk according to average PM_10_ exposure in year 2000 by selected variables, the EAGLE study, Lombardy, Italy, 2002-2005.**

|  | **PM_10_ category - median (µg/m^3^)** | **No. cases** | **No. controls** | **OR1** | **95% CI** | **OR2** | **95% CI** |
| --- | --- | --- | --- | --- | --- | --- | --- |
|  |  |  |  |  |  |  |  |
| **Men** | 1 - 40.0 | 275 | 282 | 1.00 | Reference | 1.00 | Reference |
|  | 2 - 45.4 | 292 | 276 | 1.03 | 0.72-1.46 | 1.08 | 0.75-1.57 |
|  | 3 - 47.8 | 231 | 262 | 1.00 | 0.67-1.48 | 1.07 | 0.71-1.62 |
|  | 4 - 49.4 | 236 | 280 | 1.10 | 0.73-1.67 | 1.13 | 0.73-1.75 |
|  | 5 - 51.1 | 284 | 268 | 1.46 | 0.97-2.21 | 1.69 | 1.09-2.61 |
|  | OR per 10 µg/m^3^ |  |  | 1.27 | 0.91-1.77 | 1.43 | 1.01-2.03 |
|  |  |  |  |  |  |  |  |
| **Women** | 1 - 40.0 | 56 | 80 | 1.00 | Reference | 1.00 | Reference |
|  | 2 - 45.4 | 69 | 87 | 1.51 | 0.78-2.91 | 1.46 | 0.71-3.01 |
|  | 3 - 47.8 | 62 | 97 | 1.26 | 0.62-2.56 | 1.30 | 0.60-2.82 |
|  | 4 - 49.4 | 88 | 82 | 1.85 | 0.89-3.86 | 1.91 | 0.85-4.28 |
|  | 5 - 51.1 | 72 | 94 | 1.34 | 0.63-2.82 | 1.31 | 0.58-2.97 |
|  | OR per 10 µg/m^3^ |  |  | 0.95 | 0.52-1.75 | 0.92 | 0.47-1.78 |
|  |  |  |  |  |  |  |  |
| **Residents in the** | 1 - 40.0 | 33 | 42 | 1.00 | Reference | 1.00 | Reference |
| **Milan area** | 2 - 45.4 | 186 | 224 | 1.17 | 0.65-2.12 | 1.19 | 0.63-2.25 |
| **(19 municipalities)** | 3 - 47.8 | 241 | 303 | 1.09 | 0.61-1.95 | 1.12 | 0.60-2.09 |
|  | 4 - 49.4 | 314 | 351 | 1.32 | 0.74-2.36 | 1.35 | 0.73-2.50 |
|  | 5 - 51.1 | 355 | 362 | 1.50 | 0.84-2.68 | 1.64 | 0.88-3.03 |
|  | OR per 10 µg/m^3^ |  |  | 1.38 | 0.93-2.06 | 1.51 | 0.99-2.31 |
|  |  |  |  |  |  |  |  |
| **Residents in** | 1 - 40.0 | 298 | 320 | 1.00 | Reference | 1.00 | Reference |
| **the other 4 areas** | 2 - 45.4 | 175 | 139 | 1.11 | 0.76-1.60 | 1.15 | 0.78-1.71 |
| **(197 municipalities)** | 3 - 47.8 | 52 | 56 | 0.98 | 0.57-1.67 | 1.00 | 0.56-1.76 |
|  | 4 - 49.4 | 10 | 11 | 0.86 | 0.29-2.61 | 0.81 | 0.26-2.57 |
|  | 5 - 51.1 | 1 | 0 | NC^a^ |  | NC^a^ |  |
|  | OR per 10 µg/m^3^ |  |  | 1.04 | 0.69-1.58 | 1.17 | 0.76-1.82 |
|  |  |  |  |  |  |  |  |
| **Residents** | 1 - 40.0 | 15 | 25 | 1.00 | Reference | 1.00 | Reference |
| **in the city of** | 2 - 45.4 | 118 | 170 | 1.37 | 0.61-3.09 | 1.49 | 0.60-3.69 |
| **Milan** | 3 - 47.8 | 215 | 267 | 1.53 | 0.70-3.38 | 1.70 | 0.70-4.15 |
|  | 4 - 49.4 | 273 | 312 | 1.79 | 0.81-3.92 | 2.02 | 0.83-4.90 |
|  | 5 - 51.1 | 316 | 321 | 2.05 | 0.94-4.50 | 2.47 | 1.02-5.99 |
|  | OR per 10 µg/m^3^ |  |  | 1.75 | 1.09-2.79 | 2.07 | 1.25-3.42 |
|  |  |  |  |  |  |  |  |
| **Residents** | 1 - 40.0 | 316 | 337 | 1.00 | Reference | 1.00 | Reference |
| **in the other** | 2 - 45.4 | 243 | 193 | 1.12 | 0.80-1.57 | 1.16 | 0.81-1.65 |
| **215 municipalities** | 3 - 47.8 | 78 | 92 | 0.78 | 0.50-1.22 | 0.82 | 0.51-1.32 |
|  | 4 - 49.4 | 51 | 50 | 1.02 | 0.57-1.84 | 0.87 | 0.47-1.62 |
|  | 5 - 51.1 | 40 | 41 | 1.19 | 0.60-2.33 | 1.22 | 0.59-2.49 |
|  | OR per 10 µg/m^3^ |  |  | 0.96 | 0.67-1.39 | 1.06 | 0.72-1.56 |
|  |  |  |  |  |  |  |  |
| **Never cigarette** | 1 - 40.0 | 22 | 120 | 1.00 | Reference | 1.00 | Reference |
| **smokers** | 2 - 45.4 | 26 | 106 | 2.07 | 0.86-4.99 | 2.05 | 0.79-5.34 |
|  | 3 - 47.8 | 18 | 124 | 1.68 | 0.62-4.53 | 1.61 | 0.55-4.71 |
|  | 4 - 49.4 | 23 | 108 | 2.77 | 0.99-7.75 | 2.73 | 0.90-8.35 |
|  | 5 - 51.1 | 24 | 124 | 2.61 | 0.93-7.33 | 2.62 | 0.85-8.01 |
|  | OR per 10 µg/m^3^ |  |  | 1.01 | 0.63-1.62 | 1.51 | 0.68-3.39 |
|  |  |  |  |  |  |  |  |
| **Former cigarette** | 1 - 40.0 | 151 | 157 | 1.00 | Reference | 1.00 | Reference |
| **smokers** | 2 - 45.4 | 162 | 167 | 0.97 | 0.62-1.51 | 1.05 | 0.66-1.68 |
|  | 3 - 47.8 | 135 | 149 | 1.08 | 0.66-1.76 | 1.10 | 0.66-1.82 |
|  | 4 - 49.4 | 134 | 167 | 1.17 | 0.70-1.98 | 1.19 | 0.69-2.06 |
|  | 5 - 51.1 | 142 | 145 | 1.53 | 0.90-2.60 | 1.66 | 0.95-2.89 |
|  | OR per 10 µg/m^3^ |  |  | 1.24 | 0.79-1.94 | 1.31 | 0.82-2.10 |
|  |  |  |  |  |  |  |  |
| **Current cigarette** | 1 - 40.0 | 158 | 85 | 1.00 | Reference | 1.00 | Reference |
| **smokers** | 2 - 45.4 | 173 | 90 | 1.08 | 0.64-1.79 | 1.03 | 0.60-1.78 |
|  | 3 - 47.8 | 140 | 86 | 0.88 | 0.49-1.56 | 0.94 | 0.51-1.75 |
|  | 4 - 49.4 | 167 | 87 | 1.04 | 0.57-1.88 | 1.10 | 0.58-2.08 |
|  | 5 - 51.1 | 190 | 93 | 1.18 | 0.65-2.14 | 1.39 | 0.73-2.63 |
|  | OR per 10 µg/m^3^ |  |  | 1.08 | 0.68-1.70 | 1.28 | 0.79-2.10 |

OR1, odds ratios adjusted for area, gender, age, education, and smoking (active and passive); OR2, odds ratios additionally adjusted for dietary and occupational variables. The ORs per 10 µg/m^3^ were derived from models with continuous PM_10_ concentration levels.

^a^NC: not calculated
