# Supplementary material for: Outdoor particulate matter (PM10) exposure and lung cancer risk in the EAGLE study
Source: PLoS One. 2018 Sep 14;13(9):e0203539. doi: 10.1371/journal.pone.0203539 (PMC6157824; doi:10.1371/journal.pone.0203539)
Supplement: S4 Table — (DOCX) [file pone.0203539.s004.docx]

**S4 Table. Lung cancer risk according to average PM_10_ exposure in year 2000 by histological type, the EAGLE study, Lombardy, Italy, 2002-2005.**

|  | **PM_10_ category - median (µg/m^3^)** | **No. cases** | **No. controls** | **OR1** | **95% CI** | **OR2** | **95% CI** |
| --- | --- | --- | --- | --- | --- | --- | --- |
|  |  |  |  |  |  |  |  |
| **Adenocarcinoma** | 1 - 40.0 | 134 | 362 | 1.00 | Reference | 1.00 | Reference |
|  | 2 - 45.4 | 156 | 363 | 1.02 | 0.72-1.46 | 1.11 | 0.77-1.61 |
|  | 3 - 47.8 | 126 | 359 | 0.95 | 0.64-1.41 | 1.00 | 0.65-1.51 |
|  | 4 - 49.4 | 133 | 362 | 1.07 | 0.71-1.63 | 1.14 | 0.73-1.78 |
|  | 5 - 51.1 | 135 | 362 | 1.12 | 0.74-1.71 | 1.21 | 0.77-1.89 |
|  | OR per 10 µg/m^3^ |  |  | 1.02 | 0.73-1.44 | 1.13 | 0.79-1.62 |
|  |  |  |  |  |  |  |  |
| **Squamous cell** | 1 - 40.0 | 94 | 362 | 1.00 | Reference | 1.00 | Reference |
| **Carcinoma** | 2 - 45.4 | 101 | 363 | 1.23 | 0.79-1.92 | 1.20 | 0.75-1.92 |
|  | 3 - 47.8 | 84 | 359 | 1.28 | 0.77-2.13 | 1.27 | 0.74-2.17 |
|  | 4 - 49.4 | 73 | 362 | 1.39 | 0.81-2.40 | 1.30 | 0.73-2.32 |
|  | 5 - 51.1 | 80 | 362 | 1.50 | 0.87-2.58 | 1.69 | 0.95-3.02 |
|  | OR per 10 µg/m^3^ |  |  | 1.30 | 0.84-2.03 | 1.44 | 0.90-2.29 |
|  |  |  |  |  |  |  |  |
| **Small cell** | 1 - 40.0 | 32 | 362 | 1.00 | Reference | 1.00 | Reference |
| **Carcinoma** | 2 - 45.4 | 39 | 363 | 1.23 | 0.66-2.30 | 1.02 | 0.52-1.98 |
|  | 3 - 47.8 | 29 | 359 | 1.00 | 0.49-2.05 | 1.00 | 0.48-2.11 |
|  | 4 - 49.4 | 28 | 362 | 1.12 | 0.52-2.38 | 0.90 | 0.40-2.00 |
|  | 5 - 51.1 | 42 | 362 | 1.64 | 0.79-3.42 | 1.56 | 0.72-3.38 |
|  | OR per 10 µg/m^3^ |  |  | 1.41 | 0.75-2.64 | 1.35 | 0.70-2.63 |

OR1, odds ratios adjusted for area, gender, age, education, and smoking (active and passive); OR2, odds ratios additionally adjusted for dietary and occupational variables. The ORs per 10 µg/m^3^ were derived from models with continuous PM_10_ concentration levels.
