# Supplementary figures and images for: Outdoor particulate matter (PM10) exposure and lung cancer risk in the EAGLE study
Source: PLoS One. 2018 Sep 14;13(9):e0203539. doi: 10.1371/journal.pone.0203539 (PMC6157824; doi:10.1371/journal.pone.0203539)

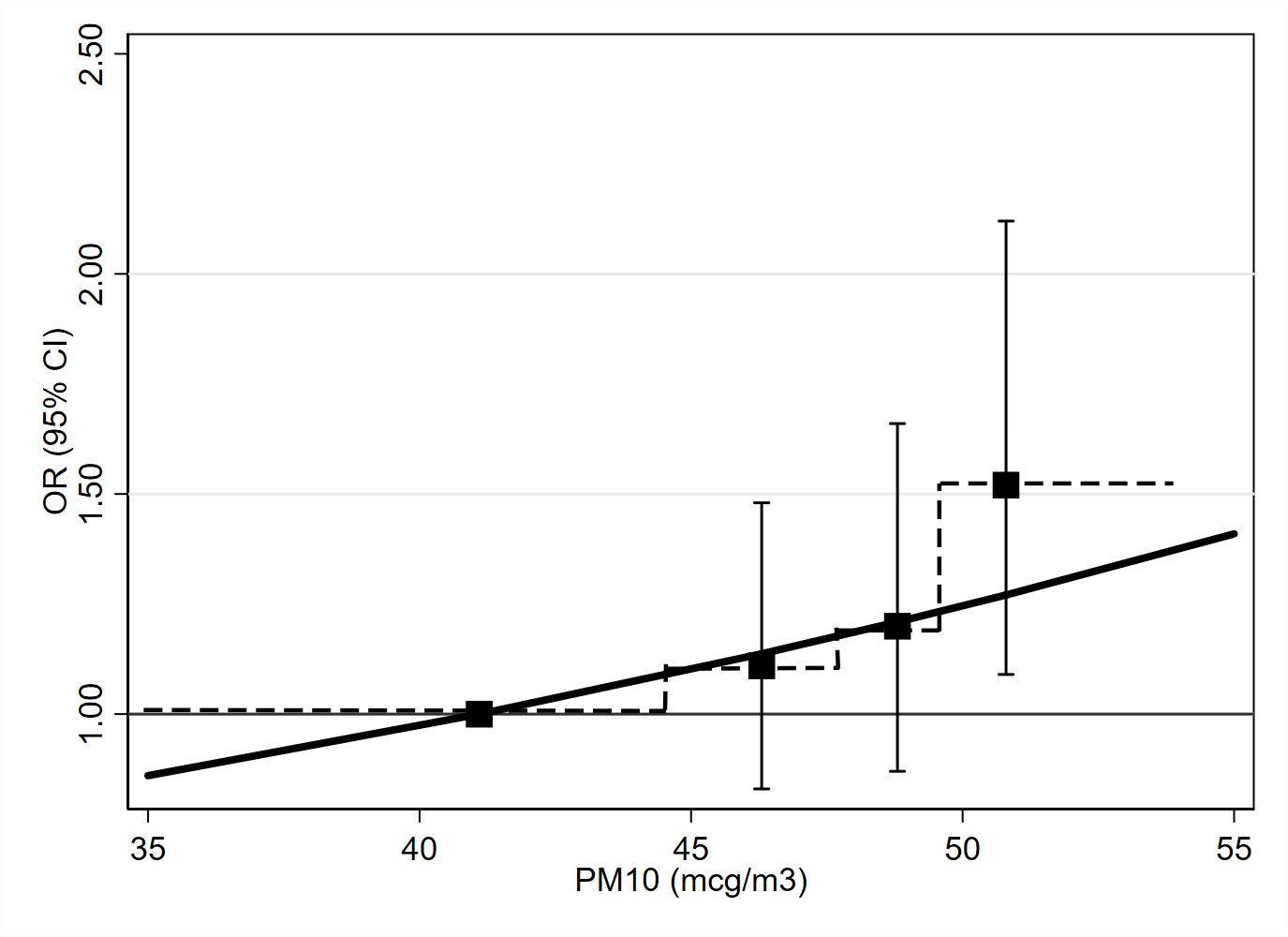

Supplement: S1 Fig — (TIF) [file pone.0203539.s005.tif]
