# Supplementary material for: Outdoor particulate matter (PM10) exposure and lung cancer risk in the EAGLE study
Source: PLoS One. 2018 Sep 14;13(9):e0203539. doi: 10.1371/journal.pone.0203539 (PMC6157824; doi:10.1371/journal.pone.0203539)
Supplement: S2 File — (DOCX) [file pone.0203539.s007.docx]

**S2 File. Legend for the minimal anonymized dataset (S1 File).**

| **Variable** | **Meaning** |
| --- | --- |
| case | Case status |
| morphogrp | Lung cancer morphology |
| stage7 | Lung cancer stage at diagnosis |
| othercancerall | Primary cancer(s) (previously or newly-diagnosed) other than lung cancer: 0=No; 1=Yes |
| area | Area of residence at enrollment: MI, MZ, BS, PV, VA |
| milan | Residence in the city of Milan; 0=No; 1=Yes |
| sex | Gender: 1=Male; 2=Female |
| education | Education |
| age_study_cat | Age category (five-year classes) at enrollment |
| age_study | Age at enrollment |
| cigt_ever | Ever smoked cigarettes: 0=No;1=Yes |
| cigt_yquitcat | Years since quitting smoking category |
| cigt_cum_packyear | Cigarette pack-years |
| smkother | Ever smoked other tobacco products: 0=No;1=Yes |
| ets_total | Ever exposed to passive smoking: 0=No;1=Yes |
| redmeat3 | Usual frequency of red meat consumption: 1=Low; 2=Medium; 3=High |
| fruitveg3 | Usual frequency of fruit and vegetable consumption: 1=Low; 2=Medium; 3=High |
| meatproc3 | Usual frequency of processed meat consumption: 1=Low; 2=Medium; 3=High |
| domasbcat3 | Ever exposed to asbestos: 0=No;1=Low exposure; 2=High exposure |
| domsilcat3 | Ever exposed to crystalline silica: 0=No;1=Low exposure; 2=High exposure |
| dompahcat3 | Ever exposed to PAH: 0=No;1=Low exposure; 2=High exposure |
| domnicrcat3 | Ever exposed to nickel/chromium compounds: 0=No;1=Low exposure; 2=High exposure |
| domdmecat3 | Ever exposed to diesel motor exhausts: 0=No;1=Low exposure; 2=High exposure |
| pm10 | Average PM10 concentration (μg/m^3^) in the year 2000 |
| pm10cat4 | Medians of four PM10 categories |
| pm10cat5 | Medians of five PM10 categories |
| radon | Probability (%) that a generic house at ground floor has a radon concentration >200 Bq/m^3^ |
